# Non-linear association between composite dietary antioxidant index and depression

**DOI:** 10.3389/fpubh.2022.988727

**Published:** 2022-10-13

**Authors:** Leiyong Zhao, Yiyan Sun, Renshuang Cao, Xueqiang Wu, Tianjiao Huang, Wei Peng

**Affiliations:** ^1^Department of Psychosomatic Medicine, Affiliated Hospital of Shandong University of Traditional Chinese Medicine, Jinan, China; ^2^The First Clinical College of Shandong University of Traditional Chinese Medicine, Jinan, China

**Keywords:** non-linear, CDAI, depression, NHANES, cross-sectional research

## Abstract

**Background:**

Growing evidence has shown that the antioxidant diet is a protective factor against depression. However, the relationship between the Composite Dietary Antioxidant Index (CDAI), an important measure of antioxidant diet, and depression has received little attention. Therefore, we investigated the relationship between CDAI and depression through a cross-sectional analysis of the National Health and Nutrition Examination Survey (NHANES) from 2007 to 2018.

**Methods:**

The association between CDAI and depression was investigated using a weighted multiple logistic regression model with subgroup analysis. Non-linear correlations were explored using fitted smoothing curves. And we used a recursive method to figure out the turning point and build a weighted two-piece linear regression model.

**Results:**

In the multivariate logistic regression model with full adjustment for confounding variables, the ORs (95% CI) for the association between CDAI and depression were 0.83 (0.78, 0.88). Moreover, a non-linear association was found, with 0.16 being the inflection point. Before the inflection point, each unit increase in CDAI was associated with a 30% decrease in the risk of depression. After the inflection point, the risk of depression was found to be reduced by 11% for each unit increase. None of the interactions in all subgroup analyses were statistically significant.

**Conclusions:**

Our study highlighted a negative non-linear association between CDAI and depression in a nationally representative sample of US adults. Further clinical and basic research is needed to explore their association better.

## Introduction

Depression is a complex group of disorders with severe dysfunction, and prolonged depression can easily lead to adverse events such as cardiovascular disease (CVD), diabetes, and stroke (1). According to the data from World Health Organization (WHO), the number of people suffering from depression worldwide is 350 million, of which about 1 million commit suicide yearly. It is expected to be the second leading cause of illness and disability worldwide by 2030 (2). For highly developed countries, like the United States, this percentage is as high as about 10%. The onset of depression has severely affected the lives of patients and their families and brought about great financial burden. Although depression has been studied intensively and extensively, its etiology, pathogenesis, and treatment have not been fully understood (3). Previous studies suggest a possible link between biomarkers of inflammation, oxidative stress, metabolic profile, and major depression (4, 5).

Oxidative stress is an imbalance between reactive oxygen species production or exposure and antioxidant defense, favoring the production of reactive oxygen species in a complex biological process (6). Numerous reports have revealed a strong relationship between hyperactive oxidative stress and depression-like behaviors (7–9). The brain is highly susceptible to oxidative stress because of its specific structure, such as high oxygen consumption, high unsaturated fatty acid content, high regional iron levels, and low antioxidant capacity. Therefore, how to effectively prevent oxidative stress has also become the focus of our attention. Results from the Kraków study suggested that daily dietary intake of antioxidants can increase antioxidant defense and reduce oxidative stress by increasing plasma levels of antioxidants (10). Adjusting the diet structure may be an effective way to alleviate depression by lowering the body's oxidative stress level.

The Composite Dietary Antioxidant Index (CDAI) is a valid and reliable nutritional tool that assesses a person's diet's overall antioxidant characteristics, which is a summary score of multiple dietary antioxidants including vitamins A, C, and E, manganese, selenium, and zinc (11). A previous cross-sectional study discovered that dietary total antioxidant capacity (DTAC) was significantly correlated with both sleep status and psychological symptoms among diabetic patients (10). However, research on the association between CDAI and depression has rarely been done. We analyzed the National Health and Nutrition Examination Survey (NHANES) database to delve into the potential association between CDAI and the onset of depression, with the aim of reducing the incidence of depression through dietary guidance.

## Materials and methods

### Study population

**Figure 1:**
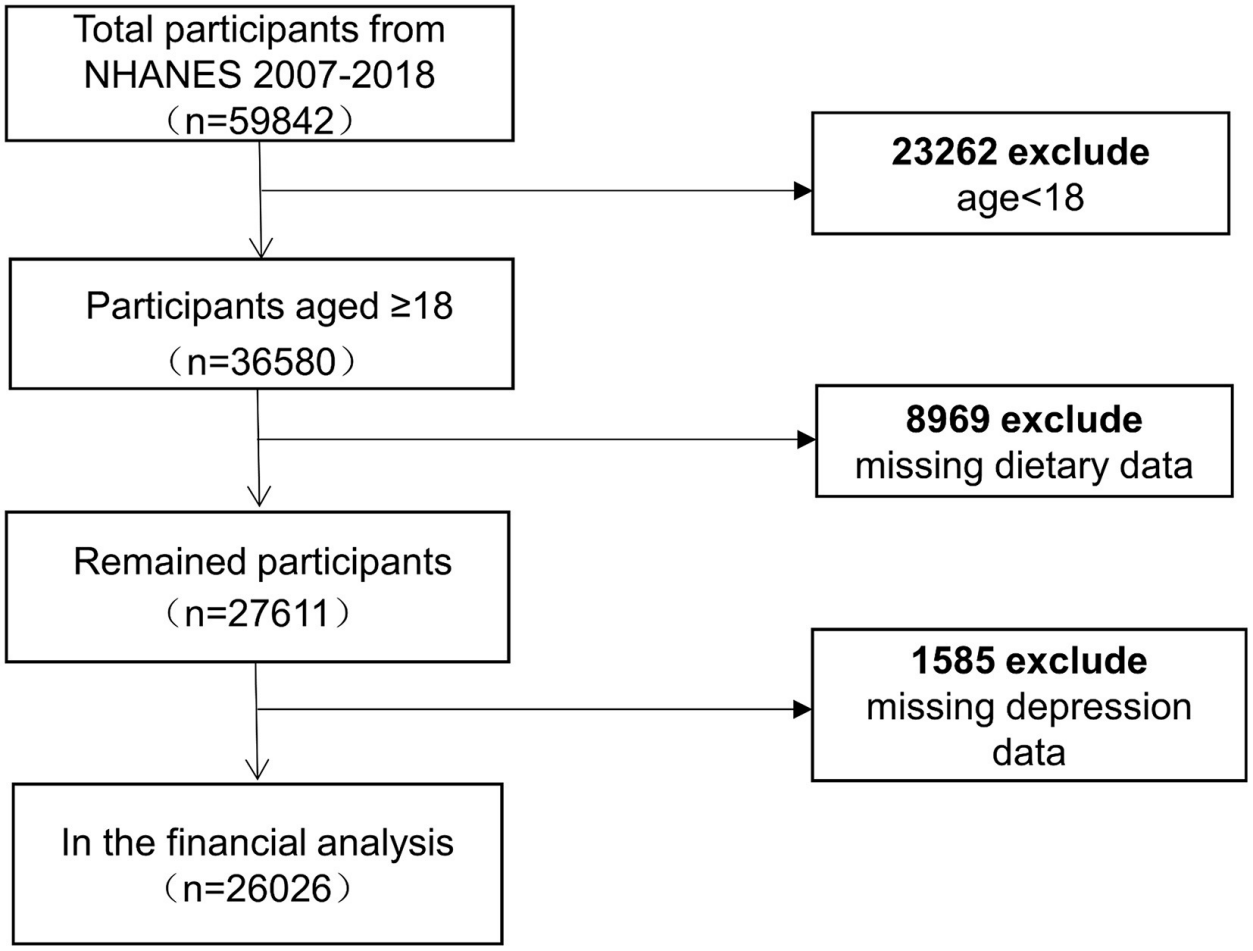
Flow chart of sample selection from the NHANES 2007–2018.

The NHANES is an ongoing survey conducted by the Center for Disease Control and Prevention (CDC) to collect information on the health, nutrition, and sociology of the US family population. After a strict inclusion of exclusion, we included six cycles with 26,026 adult participants (Figure 1). To select a representative sample of non-institutionalized US civilians, the CDC employed a multistage, complex clustered probability architecture with multiple stages. Researchers can access the survey data via the internet. The approval for protocols was granted by the National Center for Health Statistics (NCHS) ethical review board. Researchers who meet the eligibility requirements can use the database without filling out an application. All of the patient information in the database was kept anonymous. Informed permission forms were signed by participants.

### Primary exposure

The NHANES collected participants' food intake on two consecutive days via a 24-h dietary recall interview. The first dietary recall was conducted in person, and the second dietary recall proceeded 3–10 days later over the telephone. Average daily intakes were calculated from 2 days dietary recall data. The CDAI was calculated for all subjects using the measurement method recommended by Wright (11), with reference to previously published databases in the United States and Italy. We included six minerals and vitamins from food sources (manganese, selenium, zinc, vitamins A, C, and E). To calculate CDAI, we subtracted the universal mean and divided the result by its worldwide standard deviation from the six dietary minerals and vitamins as follows:


$$\begin{array}{l} \text{CDAI}=\sum_{\text{i }=\;1}^{\text{n}=\;6\;}\frac{\text{Individual Intake}-\text{ Mean}}{\text{SD}} \end{array}$$

### Outcomes

The 9-item Patient Health Questionnaire (PHQ-9) was used to assess depressive symptoms in the 2 weeks before the survey. The PHQ-9 questionnaire is designed to assess each of the nine diagnostic criteria for major depression listed in the Diagnostic and Statistical Manual of Mental Disorders, Fourth Edition (emotional depression, loss of interest, sleep disorders, fatigue, low self-esteem, appetite problems, difficulty concentrating, psychomotor agitation or retardation, and suicidal thoughts). The score for each question was defined as 0 (not at all), 1 (a few days), 2 (more than half of the days), and 3 (nearly every day), with a total scale score range of 0–27 (12). Individuals with a total PHQ-9 score of 10 are considered to have severe depression; this cutoff has 88% sensitivity and 88% specificity (12).

### Covariates

Demographic, smoking status, vigorous recreational activity, and self-reported health data were obtained using the Computer Assisted Personal Interviewing system. Demographic variables included age, sex, race/ethnicity, education level, marriage, and income to poverty ratio. The income to poverty ratio is calculated relative to the Department of Health and Human Services poverty guidelines for household income and is categorized as ≤ 130% or >130% (13). The questionnaire data included smoking status, vigorous recreational activity, stroke, CVD, cancer, and diabetes. Smoking status was categorized as “never” (lifetime < 100 cigarettes), “former” (previous history of smoking but no longer smoking at the time of the interview), or “now” (lifetime cigarette use) (14). The vigorous recreational activity was assessed using the General Practice Physical Activity Questionnaire (GPPAQ), classified into two categories: yes and no. Stroke was identified on the basis of Question MCQ160f, explicitly asking, “Have you ever been told by a doctor or other health professional that you have a stroke?” Cancer was classified as yes or no based on the participants self-reported medical history. Energy intake was determined by averaging 2 days of dietary recall. The CVD was obtained from the Medical Conditions questionnaire, which captured if participants had been told by a physician that they had coronary heart disease, congestive heart failure, or heart attack (15). Individuals who self-reported doctor-diagnosed diabetes or had fasting plasma glucose (FPG > 7.0 mmol/L) were identified to have diabetes. Latex-enhanced nephelometry was used to measure the C-reactive protein (CRP) and uric acid in a blood sample. The body mass index (BMI) was calculated by taking a person's weight in kilograms and multiplying it by their height in meters.

### Statistical analysis

As required by the NCHS, we weighted the data in our analysis. Participants were separated into two groups based on whether they had depression. We presented continuous variables as means (standard error of magnitude, SEM) and categorical variables as quantities (percentages) in the baseline characteristics of the study population. The baseline characteristics were compared using the weighted linear regression for continuous variables and the weighted chi-square test for categorical variables. To test the relationship between CDAI and depression, we used multiple regression equations, built crude and adjusted models, and carried out tests for linear trends to ascertain the relationships consistency. Generalized additive models were then performed to assess whether there were any non-linear correlations. After detecting non-linearity, the turning point was calculated using a recursive algorithm, and then a two-piecewise linear regression model was constructed. We performed subgroup analyses and interactions to explore whether there were other relevant risk factors that might influence the association of CDAI with depression. R (version 3.5.3) and the EmpowerStats software (http://www.empowerstats.com) were used to conduct statistical analyses. *P*-values < 0.05 were considered statistically significant.

## Results

### Characteristics of the study population

The mean age of the study population was 47.35 ± 17.44, with the non-depressed population being older on average than the depressed population (46.55 ± 16.17), and the percentage of women in the depressed population was 64.54%, higher than the 35.46% of men. Income to poverty ratio, uric acid, energy, and vigorous recreational activity were lower in depressed patients than in non-depressed patients. In terms of smoking status, 60.43% of depressed patients had a history of smoking or were currently smoking (Table 1).

**Table 1:** Characteristics of the study population from NHANES 2007–2018.

	**Overall**	**No depression**	**With depression**	***P*-value**
	**(*n* = 26,026)**	**(*n* = 23,660)**	**(*n* = 2,366)**	
Age (years)	47.35 ±17.44	47.42 ± 17.55	46.55 ± 16.17	0.0307
**Sex** (%)				< 0.0001
Male	47.14	48.15	35.46	
Female	52.86	51.85	64.54	
**Race/Ethnicity** (%)				< 0.0001
Mexican American	8.05	8.05	7.97	
White	68.46	68.91	63.14	
Black	11.01	10.81	13.29	
Other	12.49	12.22	15.60	
**Education level (%)**				< 0.0001
Less than high school	14.90	13.98	24.97	
High school	23.71	23.39	27.47	
More than high school	61.39	62.63	47.55	
**Marital status (%)**				< 0.0001
Married/Living with partner	62.30	63.66	46.52	
Divorced/Separated/Widowed	17.75	16.71	29.84	
Never married	19.95	19.63	23.64	
**Vigorous recreational activities (%)**				< 0.0001
Yes	26.00	27.23	11.69	
No	74.00	72.77	88.31	
**Smoking status (%)**				< 0.0001
Now	18.02	16.30	37.93	
Former	24.91	25.12	22.49	
Never	56.07	58.58	39.58	
**Stroke (%)**				< 0.0001
Yes	2.92	2.59	6.78	
No	97.08	97.41	93.23	
**Cancer (%)**				0.1048
Yes	14.07	14.10	13.71	
No	85.93	85.90	86.29	
**Diabetes (%)**				< 0.0001
Yes	10.84	10.32	16.82	
No	89.16	89.68	83.18	
**CVD (%)**				< 0.0001
Yes	8.68	8.00	16.50	
No	91.32	92.00	83.50	
**Income to poverty ratio**	2.99 ± 1.59	3.06 ± 1.58	2.18 ± 1.50	< 0.0001
BMI	29.18 ± 6.92	29.03 ± 6.78	30.87 ± 8.17	< 0.0001
CPR	2.21 ± 4.28	2.16 ± 4.13	2.78 ± 5.70	< 0.0001
Energy	2048.73 ± 752.35	2056.96 ± 747.26	1953.22 ± 802.92	< 0.0001
Uric acid (mg/dl)	5.42 ± 1.38	5.43 ± 1.38	5.33 ± 1.40	0.0018
CDAI	0.52 ± 3.19	0.61 ± 3.17	−0.48 ± 3.23	< 0.0001

Mean ± SD for continuous variables: *P*-value was calculated by weighted linear regression.

% for categorical variables: *P*-value was calculated by weighted chi-square test.

CVD, cardiovascular disease; BMI, body mass index; CRP, c-reactive protein; CDAI, composite dietary antioxidant index.

### Association between CDAI and depression

Three models were constructed to examine the relationship between CDAI and depression in this study. Model 1, no covariate was adjusted; Model 2 was adjusted for age, gender, and race. Model 3 added to model 2 the education level, income to poverty ratio, marital status, smoking status, vigorous recreational activity, BMI, CRP, stroke, cancer, diabetes, CVD, energy, and uric acid as covariates. In the crude model, the ORs (95% CI) were 0.77 (0.74, 0.80), which indicated that the risk of depression was reduced by 23% for every unit rise in CDAI. The risk of depression dropped by 20% [0.80 (0.77, 0.84)] for each unit rise in CDAI in the model only adjusted for demographic data (model 2). In the fully adjusted model (model 3), the risk of depression decreased by 17% for each unit increase in CDAI [0.83 (0.78, 0.88)] (Table 2). For the sensitivity analysis, we converted the CDAI from a continuous variable to a quadratic categorical variable for trend testing and obtained consistent findings (Table 2).

**Table 2:** Association of composite dietary antioxidant index and depression.

	**Model 1 OR (95% CI)**	**Model 2 OR (95% CI)**	**Model 3 OR (95% CI)**
CDAI. Z score	0.74 (0.70, 0.77)	0.78 (0.74, 0.81)	0.78 (0.73, 0.84)
Lowest quartiles	1.0	1.0	1.0
2nd	0.71 (0.64, 0.80)	0.74 (0.66, 0.82)	0.79 (0.70, 0.89)
3rd	0.55 (0.49, 0.62)	0.59 (0.53, 0.67)	0.66 (0.57, 0.76)
4th	0.47 (0.41, 0.53)	0.53 (0.47, 0.61)	0.58 (0.48, 0.69)
*P* for trend	< 0.001	< 0.001	< 0.001
**Sex**			
Male	0.78 (0.73, 0.84)	0.78 (0.72, 0.84)	0.77 (0.69, 0.86)
Female	0.78 (0.73, 0.83)	0.77 (0.73, 0.82)	0.80 (0.73, 0.88)
**Race/Ethnicity**			
Mexican American	0.69 (0.61, 0.78)	0.77 (0.68, 0.88)	0.69 (0.56, 0.85)
White	0.68 (0.63, 0.73)	0.71 (0.66, 0.76)	0.83 (0.74, 0.92)
Black	0.93 (0.85, 1.02)	0.98 (0.89, 1.08)	0.90 (0.78, 1.05)
Other	0.69 (0.62, 0.76)	0.73 (0.66, 0.81)	0.73 (0.62, 0.86)
**Age**			
< 40	0.75 (0.69, 0.81)	0.79 (0.73, 0.86)	0.77 (0.68, 0.87)
40–60	0.70 (0.65, 0.75)	0.74 (0.68, 0.80)	0.84 (0.75, 0.94)
>60	0.76 (0.69, 0.83)	0.80 (0.73, 0.87)	0.72 (0.63, 0.83)
**BMI**			
< 18.5	0.81 (0.60, 1.09)	0.77 (0.57, 1.06)	0.94 (0.51, 1.73)
18.5–24.9	0.77 (0.69, 0.85)	0.78 (0.70, 0.87)	0.86 (0.73, 1.02)
25–28	0.66 (0.59, 0.73)	0.68 (0.61, 0.76)	0.64 (0.55, 0.76)
>28	0.77 (0.73, 0.82)	0.82 (0.77, 0.88)	0.81 (0.74, 0.89)

Model 1: no covariates were adjusted.

Model 2: age, sex, and race were adjusted.

Model 3: age, sex, race, income to poverty ratio, education level, marital status, smoking status, vigorous recreational activity, BMI, CRP, stroke, cancer, diabetes, CVD, energy, and uric acid were adjusted.

In the subgroup analysis stratified by age, race, BMI, or sex, the model is not adjusted for the stratification variable itself.

CDAI, composite dietary antioxidant index; BMI, body mass index.

**Figure 2:**
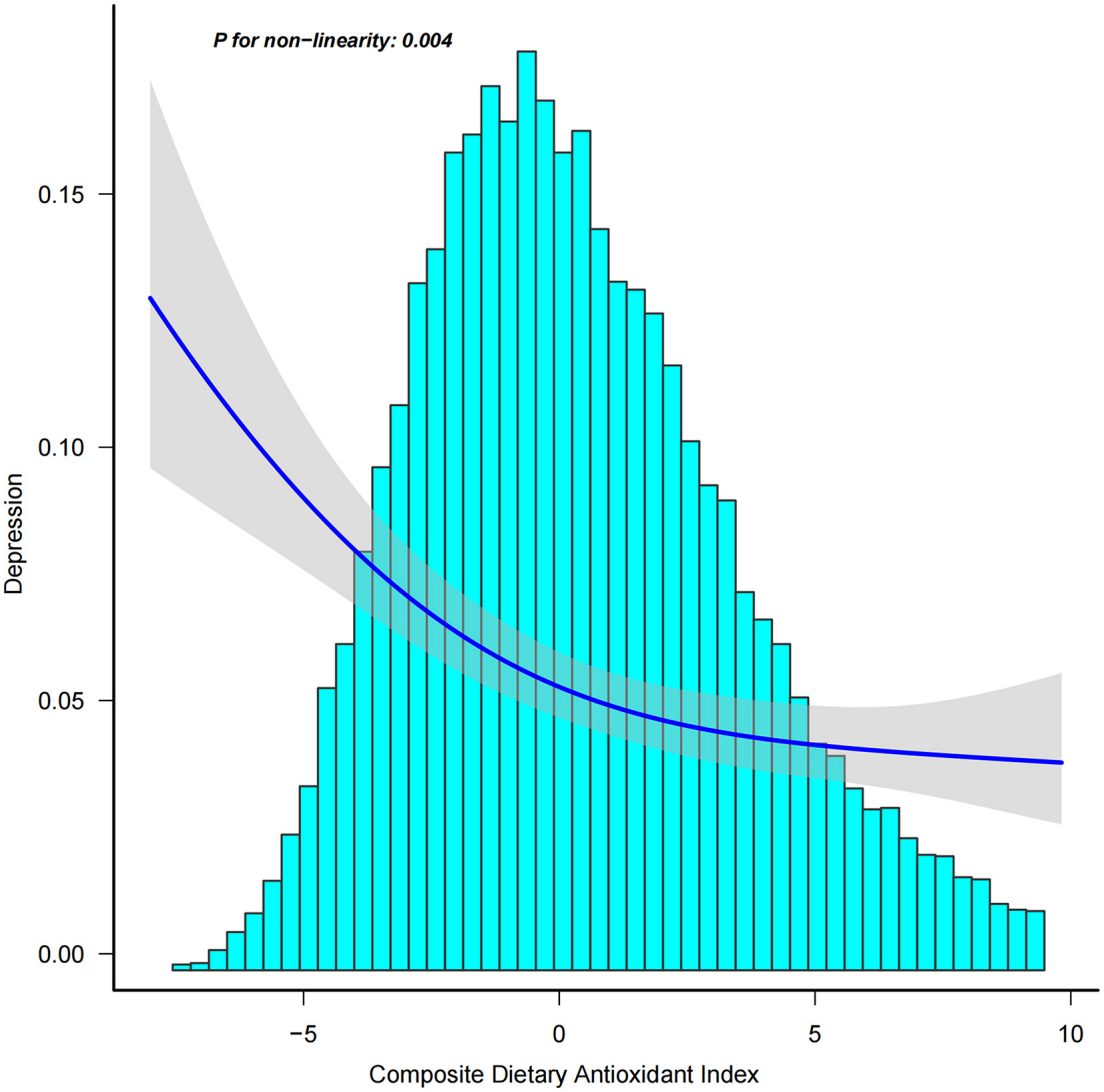
Association between CDAI and depression. Age, sex, race, income to poverty ratio, education level, marital status, smoking status, vigorous recreational activity, BMI, CRP, stroke, cancer, diabetes, CVD, energy, and uric acid were adjusted.

**Figure 3:**
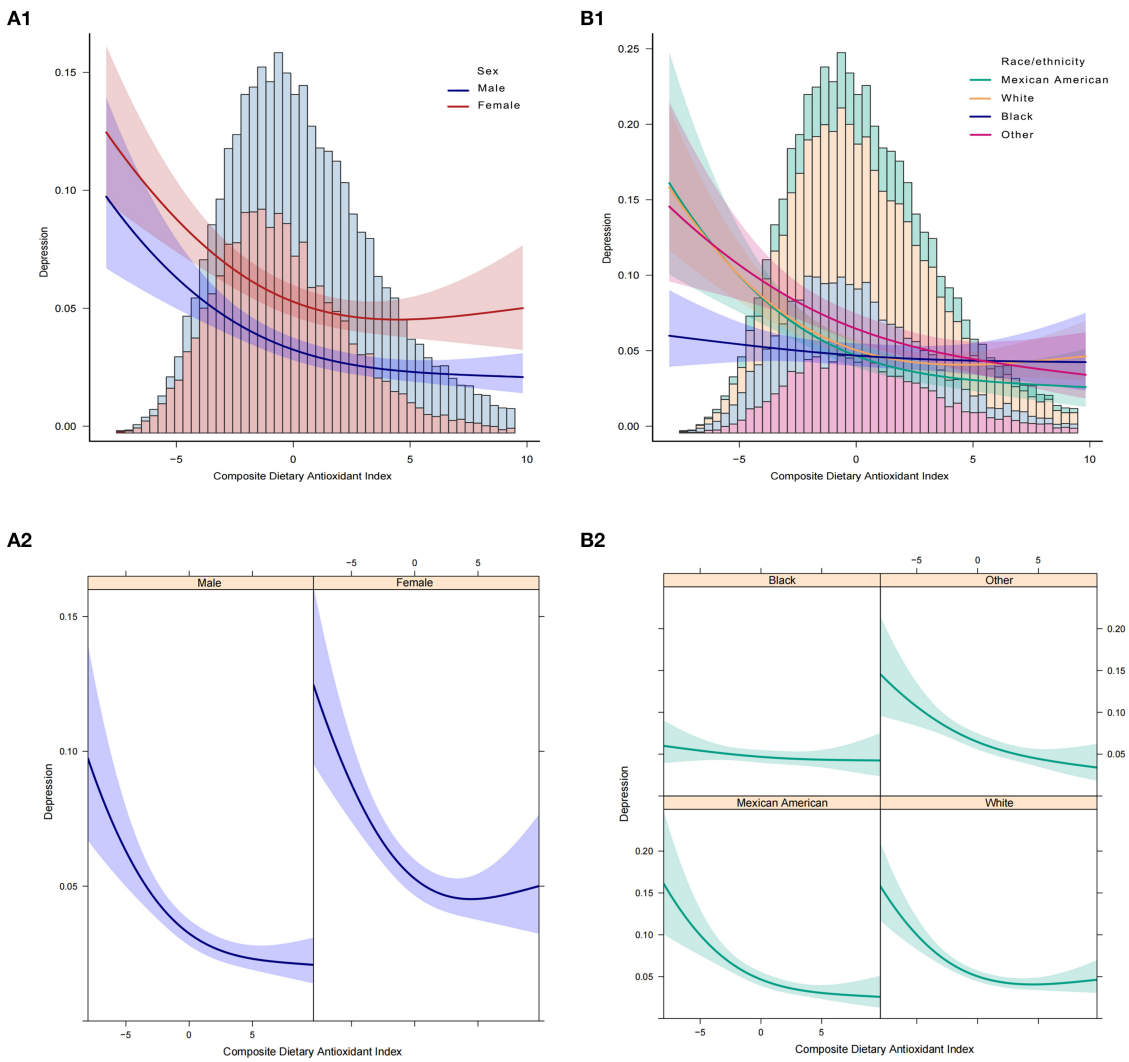
Association between CDAI and depression. **(A1,A2)** Participants stratified by sex. **(B1,B2)** Participants stratified by race. In the subgroup analysis stratified by sex or race, the model is not adjusted for sex or race, respectively.

**Figure 4:**
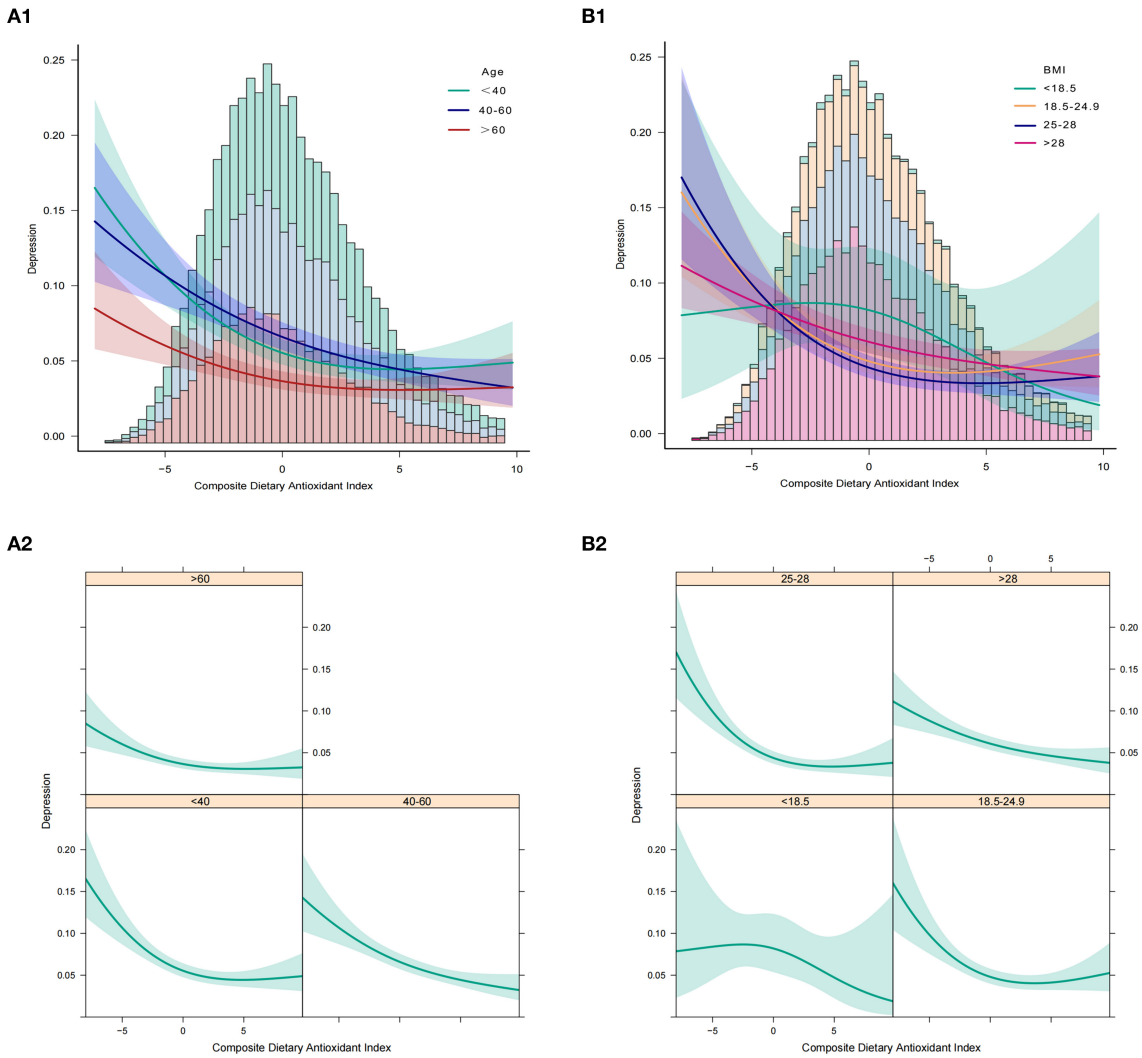
Association between CDAI and depression. **(A1,A2)** Participants stratified by age. **(B1,B2)** Participants stratified by BMI. In the subgroup analysis stratified by age or BMI, the model is not adjusted for age or BMI, respectively.

In addition, we analyzed whether there was a non-linear correlation between CDAI and depression. A smooth curve with a saturation effect was found after adjusting for age, sex, race, income to poverty ratio, education level, marital status, smoking status, vigorous recreational activity, BMI, CRP, stroke, cancer, diabetes, CVD, energy, and uric acid (*P* for non-linearity = 0.004) (Figure 2). Furthermore, we found that 0.16 was the inflection point. Significant associations were found with the ORs (95% CI) of 0.70 (0.63, 0.77) after the inflection point and with the ORs (95% CI) of 0.89 (0.80, 1.00) before the inflection point (Table 3). In the stratified curve fittings, all curves were consistent with the unstratified results except for black, those with BMI < 18.5 and >28 (Figures 3, 4).

**Table 3:** Threshold effect analysis of composite dietary antioxidant index on depression using a two-piecewise linear regression model.

**Depression**	**Adjust OR (95% CI)**	***P***
CDAI		
Fitting by standard linear model	0.78 (0.73, 0.84)	< 0.0001
**Fitting by two-piecewise linear model**		
Inflection point	0.16	
< 0.16	0.70 (0.63, 0.77)	< 0.0001
>0.16	0.89 (0.80, 1.00)	0.0437
Log-likelihood ratio	0.002	

Age, sex, race, income–poverty ratio, education level, marital status, smoking status, vigorous recreational activity, BMI, CRP, stroke, cancer, diabetes, CVD, energy, and uric acid were adjusted.

**Figure 5:**
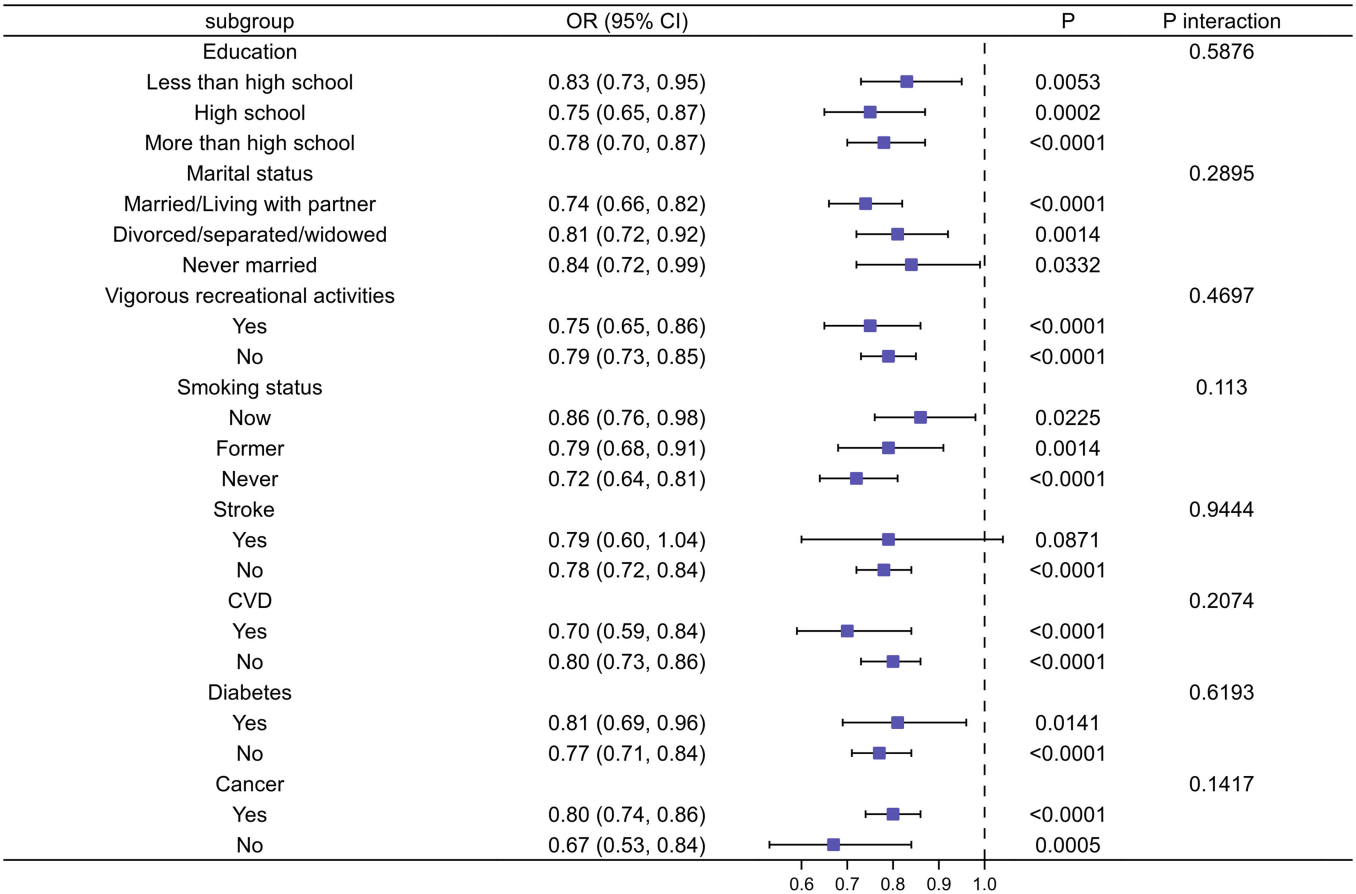
Subgroup analysis of risk factors for the relationship between CDAI and depression. In the subgroup analysis stratified by education, marital status, vigorous recreational activities, smoking status, stroke, CVD, diabetes, and cancer, the model is not adjusted for education, marital status, vigorous recreational activities, smoking status, stroke, CVD, diabetes, and cancer, respectively.

We performed subgroup analyses to explore the potential association between CDAI and the risk of depression in different populations based on education level, marital status, vigorous recreational activities, smoking status, stroke, diabetes, cancer, and CVD. The results showed that CDAI did not show statistically significant differences in reducing the risk of depression in people with a history of stroke, diabetes, CVD, cancer, and smoking, respectively, compared with the normal population. Similarly, the same conclusion was reached in different populations of education level, marital status, and vigorous recreational activities (Figure 5).

## Discussion

To the best of our own knowledge, this is the first study with a large sample exploring the relationship between CDAI and the odds of depression. We found a negative association between CDAI and depression after adjusting for all potential covariates, which indicated that CDAI was a protective factor for the development of depression. The trend of increasing CDAI with lower levels of depression appeared significantly. We performed a smoothed curve fitting and found that this negative correlation was non-linear, with 0.16 being the inflection point. Before the inflection point, each unit increase in CDAI was associated with a 30% decrease in the risk of depression. After the inflection point, the risk of depression was found to be reduced by 11% for each unit increase, and the difference between before and after the inflection point was statistically significant.

Although existing research on the relationship is still relatively rare, the use of dietary antioxidant properties to intervene in the clinical treatment of depression has long been a hot topic of research today. An analysis study of a small sample of Iranian females aged 14–16 showed a significant inverse relationship between DAI and depression (16). In a cross-sectional survey of Iran, an inverse association was found between DTAC and depression in postmenopausal women (17). A study of a male college population showed the increasing intake of vitamin E, an important antioxidant, significantly reduced the prevalence of depression (18), which is consistent with the results of research in Australian older men (19). Results from the general Danish population suggested that significant associations were observed between higher levels of the antioxidant uric acid and a lower incidence of depression as well as the usage of antidepressant medication (20).

Previous studies have shown that DTAC is negatively associated with depression, anxiety, and specific biomarkers of oxidative stress (17, 21). This result is consistent with our findings that the mechanism is closely related to oxidative stress. Patients with depression have been reported to have higher oxidative stress and inflammation levels as well as a relatively low intake of dietary antioxidants (22–24). For people with depression, antioxidant food sources are important. Many dietary antioxidants can reduce oxidative stress by using their bioactive molecules to exert antioxidant effects, integrate processes, and control gene expression in oxidative stress as cellular signaling regulators (25–27). Zinc, selenium, and manganese were essential antioxidants in protecting against oxidative stress (28, 29). Selenium is bound to selenoproteins (glutathione peroxidase and thioredoxin reductase) and prevents lipid peroxidation and oxidative cell damage (30). Consistent with this, streptozotocin-induced depressive-like behavior, oxidative stress, and neuroinflammatory responses were all decreased by the selenium compound 1-methyl-3-(phenylselany1)-1H-indole (31). Manganese is an important component of MnSOD, an antioxidant mitochondrial metalloenzyme that is protective of cells against oxidative stress (32). Zinc helps to alleviate depression by modulating glutamatergic neurotransmission and serotonergic systems, especially 5-HT1A receptor activity (33, 34). Non-enzymatic antioxidants such as vitamins A, C, and E, as well as enzymatic antioxidants such as glutathione peroxidase and superoxide dismutase, play an important role in reducing stress-induced changes in oxidants (35–37). So, antioxidant nutrients from food may be able to prevent and lessen the onset of depression caused by oxidative stress. However, the exact molecular mechanisms are not well-understood, and more research is needed to fill the gaps in this area.

According to the STROBE statement, we conducted subgroup analysis to make better use of data to reveal underlying truths. In the analysis stratified by sex, we found no significant gender differences in the relationship between CDAI and depression. This relationship was not significant among blacks, but it was stronger among Mexican Americans. The risk of depression decreased the most as the CDAI increased in the elderly population when stratified by age. In addition, we observed an independent correlation only in the overweight and obese populations. And the relationship was strongest in the BMI range of 25–27.9. When we performed stratified smooth curve fittings, we found that the relationship was consistent with a non-linear correlation for all groups except for blacks, those with BMI < 18.5, and >28. Moreover, there were no significant interactions between CDAI and other relevant risk factors, which indicated that no other factors had been identified to influence the association between CDAI and depression.

There are several limitations to this study: (1) Due to the long time span of this study and the complex way in which the CDAI was constructed, the diet assessment might involve measurement errors and inaccuracies. (2) Bias is inevitable in cross-sectional studies, and we will conduct a large cohort study in the future for further accurate evidence. (3) Since the population observed in this study was Americans, excluding special groups such as minors, we were unable to analyze special populations or other races due to the limited sample size. Therefore, further studies are needed to determine whether those findings apply universally.

## Conclusion

In conclusion, this cross-sectional study based on six cycles (2007–2018) of data from the NHANE database detected a non-linear negative association between CDAI and depression in the US adult population, after being adjusted for potential confounders. The present study provides a new avenue to explore the factors influencing dietary interventions for depression to reduce its incidence. In the future, additional randomized controlled trials or cohort studies are urgently needed to confirm this finding to provide more accurate and effective prevention and treatment options for depression prevention.

## Data availability statement

The original contributions presented in the study are included in the article/supplementary material, further inquiries can be directed to the corresponding author/s.

## Ethics statement

Written informed consent was obtained from the individual(s) for the publication of any potentially identifiable images or data included in this article.

## Author contributions

LZ: project development and research design and manuscript writing. WP and TH: responsibility for data analysis and interpretation and manuscript writing. RC and YS: manuscript editing and interpretation of the results. XW: responsibility for data collection. All authors contributed to the article and approved the submitted version.

## Funding

This work was supported by Shandong Provincial Natural Science Foundation Major Basic Research Project (Grant Number ZR2020ZD17) and Shandong Province Chinese Medicine Science and Technology Project (Grant Number 2021M150).

## Conflict of interest

The authors declare that the research was conducted in the absence of any commercial or financial relationships that could be construed as a potential conflict of interest.

## Publisher's note

All claims expressed in this article are solely those of the authors and do not necessarily represent those of their affiliated organizations, or those of the publisher, the editors and the reviewers. Any product that may be evaluated in this article, or claim that may be made by its manufacturer, is not guaranteed or endorsed by the publisher.
